# Acute hypoxia diminishes the relationship between blood pressure and subarachnoid space width oscillations at the human cardiac frequency

**DOI:** 10.1371/journal.pone.0172842

**Published:** 2017-02-27

**Authors:** Magdalena Wszedybyl-Winklewska, Jacek Wolf, Ewa Swierblewska, Katarzyna Kunicka, Agnieszka Gruszecka, Marcin Gruszecki, Wieslawa Kucharska, Pawel J. Winklewski, Joanna Zabulewicz, Wojciech Guminski, Michal Pietrewicz, Andrzej F. Frydrychowski, Leszek Bieniaszewski, Krzysztof Narkiewicz

**Affiliations:** 1 Institute of Human Physiology, Medical University of Gdansk, Gdansk, Poland; 2 Department of Hypertension and Diabetology, Medical University of Gdansk, Gdansk, Poland; 3 Department of Radiology Informatics and Statistics, Medical University of Gdansk, Gdansk, Poland; 4 Institute of Health Sciences, Pomeranian University of Slupsk, Slupsk, Poland; 5 Department of Computer Communications, Faculty of Electronics, Telecommunications and Informatics, Gdansk University of Technology, Gdansk, Poland; 6 Department of Biomedical Engineering, Faculty of Electronics, Telecommunications and Informatics, Gdansk University of Technology, Gdansk, Poland; 7 Centre for Medical Simulation, Medical University of Gdansk, Gdansk, Poland; Nagoya University, JAPAN

## Abstract

**Background:**

Acute hypoxia exerts strong effects on the cardiovascular system. Heart-generated pulsatile cerebrospinal fluid motion is recognised as a key factor ensuring brain homeostasis. We aimed to assess changes in heart-generated coupling between blood pressure (BP) and subarachnoid space width (SAS) oscillations during hypoxic exposure.

**Methods:**

Twenty participants were subjected to a controlled decrease in oxygen saturation (SaO_2_ = 80%) for five minutes. BP and heart rate (HR) were measured using continuous finger-pulse photoplethysmography, oxyhaemoglobin saturation with an ear-clip sensor, end-tidal CO_2_ with a gas analyser, and cerebral blood flow velocity (CBFV), pulsatility and resistive indices with Doppler ultrasound. Changes in SAS were recorded with a recently-developed method called near-infrared transillumination/backscattering sounding. Wavelet transform analysis was used to assess the relationship between BP and SAS oscillations.

**Results:**

Gradual increases in systolic, diastolic BP and HR were observed immediately after the initiation of hypoxic challenge (at fifth minute +20.1%, +10.2%, +16.5% vs. baseline, respectively; all P<0.01), whereas SAS remained intact (P = NS). Concurrently, the CBFV was stable throughout the procedure, with the only increase observed in the last two minutes of deoxygenation (at the fifth minute +6.8% vs. baseline, P<0.05). The cardiac contribution to the relationship between BP and SAS oscillations diminished immediately after exposure to hypoxia (at the fifth minute, right hemisphere -27.7% and left hemisphere -26.3% vs. baseline; both P<0.05). Wavelet phase coherence did not change throughout the experiment (P = NS).

**Conclusions:**

Cerebral haemodynamics seem to be relatively stable during short exposure to normobaric hypoxia. Hypoxia attenuates heart-generated BP SAS coupling.

## Introduction

Acute normobaric hypoxia exerts strong effects on the control of the cardiovascular system. Hypoxic stimuli via peripheral chemoreceptors lead directly to activation of the sympathetic nervous system and indirectly to vagal withdrawal, both of which contribute to tachycardia. As the left ventricle stroke volume remains unchanged, the net result is an increase in cardiac output [1]. Higher cardiac output and blood pressure (BP) lead to augmented cerebral blood flow (CBF), cerebral blood volume and subsequently to elevated intracranial pressure (ICP) [2,3]. Furthermore, hypoxia augments concentrations of hypoxia-inducible transcription factor, circulating pro-inflammatory cytokines and reactive oxygen species, with subsequent vascular leakage and enhanced blood-brain barrier permeability [4,5]. Finally, hypoxia increases the expression of corticotrophin releasing factor, corticotrophin releasing factor receptor type 1, aquaporin-4, and endothelin-1 in the brain [6,7]. All of these changes contribute to relatively rapid development of discrete brain oedema. The brain parenchyma is effectively protected from the detrimental influences of the augmented ICP, as the cerebral spinal fluid (CSF) can be shifted from the cranium externally to the high-compliance compartment in the spine [8,9]. An increase in cerebral blood volume, brain parenchyma volume and displacement of CSF to the spinal area have been observed using magnetic resonance imaging after only 20 minutes of exposure to acute hypoxia [2].

Being limited by a rigid skull, the complex intracranial fluid regulatory mechanism evolved to manage augmented blood flow during cardiac systolic phase. The transient increase in blood volume entering the cranium during cardiac systolic phase is compensated by displacement of an approximately equal amount of CSF through the foramen magnum into the spinal column [10]. A sophisticated windkessel mechanism, including CSF and cerebral venous system, acts as an immediate buffer for blood during the ejection phase of the heart to smooth blood flow through the cerebral capillary bed [11]. Circulating CSF between the brain and spinal cord compartments is reflected in corresponding changes in the subarachnoid space width (SAS) [8,9]. Interestingly, pulsatile CSF motion, including flow reversal in the aqueduct as well as the associated moderate changes in ICP, are increasingly recognised as a key factor ensuring proper brain haemodynamics and homeostasis [9]. Abnormal arterial/venous pulsatility has been termed pulse wave encephalopathy [12], while pulse wave injury has been associated with early disruption to the structural properties of white matter [13]. Very recently, an elevated aqueductal CSF pulsatility has been linked to white matter changes in subjects suffering from arterial hypertension [14].

Recently, our group developed a non-invasive method based on infrared radiation to assess instantaneous changes in the SAS, called near-infrared transillumination/backscattering sounding (NIR-T/BSS) [7,8]. NIR-T/BSS uses the SAS filled with translucent CSF as a propagation duct for infrared radiation [15,16]. SAS changes measured with magnetic resonance imaging and NIRT-B/SS demonstrated high interdependence between both methods (r  =  0.81, p<0.001) [17]. Furthermore SAS decline has been linked to cerebral blood volume increases during both apnoea, and acetazolamine challenge [18,19,20]. The high sampling frequency (70 Hz) of NIR-T/BSS allows for signal analysis up to 35 Hz according to Nyquist theorem. We decided to analyse collected signals up to 5 Hz due to lack any physiological process over this frequency. The power spectrum density of SAS oscillations shows clear peaks at the cardiac frequency (f0) and its harmonics (f1, f2, f3) [15,21]. Thus, using NIR-T/BSS one may accurately register rapid SAS oscillations secondary to systolic-diastolic changes in blood volume of cerebral circulation [21]. Calculating wavelet phase and amplitude coherences one may identify similarity of two oscillators. Combining NIR-T/BSS recordings and mathematical modelling, a proportion of systemic BP oscillations with corresponding heart-driven SAS changes may be precisely identified. Coupling functions enabled us to unveil a new perspective on how the neurophysiological mechanisms are affected by various stimuli. In particular we have shown that coherence of BP and SAS amplitudes decrease during apnoea in normal healthy subjects [21] but remain stable in professional apnoea divers [22]. Quite surprisingly sympathetic nervous system appears to stabilize the above mentioned relationship [22,23]. On the contrary, changes in intrathoracic pressure evoke large swings in BP and SAS amplitudes coherence (submitted to PLoS One, PONE-D-16-33950R1).

There are several commonly encountered chronic diseases which are associated with either continuous or pulsatile hypoxia. Last two decades clearly showed that widely prevalent obstructive sleep apnea (OSA) plays a substantial role in the pathogenesis of cardiovascular disease e.g. hypertension, cardiac arrhythmias or ischemic heart disease. Although reoccurring sleep-apneic episodes are associated with several detrimental phenomena, the hypoxic stimulus appears to be of critical importance for the cardiovascular morbidity [24–26]. Each apnea is characterized by a different time-interval, however, the duration of apneic events averages at 30 seconds (s) in unselected OSA population, but it may last up to several minutes in most severe cases [27,28]. Such hypoxic stimuli reoccur hundreds of times during a single night which holds true for untreated, severe OSA. In our previous experiments we have investigated the effects of typical for OSA stimuli like apnoea [21,22], negative thoracic pressure (submitted to PLoS One, PONE-D-16-33950R1) and sympathetic excitation on BP SAS interactions [23]. In this study we focused on hypoxia. The reduction in CSF volume within the brain compartment described by Dubowitz et al. [2] was only 3 to 5 ml after 20 minutes of hypoxic exposure. Therefore, we hypothesised that exposure to hypoxia for 5 minutes would be too short to affect the SAS. However, we did expect changes in the heart-generated relationship between BP and SAS oscillations.

## Materials and methods

### Ethical approval

The study conformed to the standards set by the Declaration of Helsinki. The experimental protocol and the study design were approved by the Ethics Committee of the Medical University of Gdansk (NKEBN/48/2011). All subjects gave written informed consent to participate in the study. The subjects were told not only of the procedures and risks of the experiments but also that they were free to withdraw at any time without jeopardy. The experiments were conducted by suitably qualified medical personnel.

### Subjects

Experiments were performed in a group of 20 (6 females) healthy, non-smoking volunteers (Table 1). Nicotine, coffee, tea, cocoa and methylxanthine-containing food and beverages were not permitted for 8 hours before the tests. Additionally, prior to each test, the volunteers were asked to rest comfortably for 30 minutes in the supine position.

**Table 1:** Characteristics of the study participants. Data are presented as mean values and standard deviations (SD).

	Males (n = 14)	Females (n = 6)
Age (years)	21.1±2.0	21.5±2.1
BMI (kg*m^-2^)	23.4±2.7	21.0±1.1
cc-TQR (AU)	173.6±59.5	150.8±105.9
cc-TQL (AU)	90.9±53.9	102.4±76.4
sas-TQR (AU)	824.3±250.1	992.6±241.3
sas-TQL (AU)	569.6±176.8	676.4±119.4
SBP (mmHg)	119.0±6.0	109.8±8.3
DBP (mmHg)	73.6±10.2	72.2±5.3
HR (beats*s^-1^)	64.6±9,0	72.2±9,5
CBFV (cm*s^-1^)	38.2±6.9	47.4±11.6
RI	0.6±0.1	0.6±0,1
PI	1.65±0,45	1.44±0.73
SaO_2_ (%)	97.4±1.6	96.4±1.8

sas-TQ—slow component of the subarachnoid width (<0.05 Hz); cc-TQ—cardiac component of the subarachnoid width (heart-generated pial artery pulsation, from 0.5 Hz to 5.0 Hz); SBP—systolic blood pressure; DBP—diastolic blood pressure; HR—heart rate; CBFV—cerebral blood flow velocity; PI—pulsatility index; RI—resistive index; SaO2—oxyheamoglobin saturation; kg—kilograms; m—meters; AU—arbitrary units; mmHg—millimeters of mercury; s—seconds

### Experimental design

**Fig 1:**
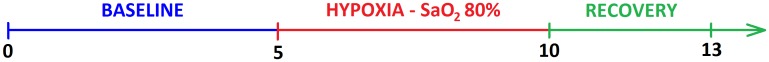
Schematic representation of the study design.

Participants were instrumented with a full-face mask connected to a one-way valve, inspiratory gas mixing reservoir, and expiratory tube with an EtCO_2_ sensor. After baseline measurements had been obtained, participants were subjected to a controlled hypoxic challenge. The procedure was as follows: the room-air was progressively saturated with an increasing content of atmospheric nitrogen so that the percentage of inhaled oxygen was gradually diminished. Participants were titrated with a mixture of breathing gases to reach SaO_2_ around 80%. Blood oxygen desaturation was maintained during the next 5 minutes, followed by 3 minutes of recovery (undisturbed breathing in room air). A graphical presentation of the study design is shown in Fig 1. For wavelet transform analysis, 10 s data windows were taken from baseline and from the end of each one-minute time series of hypoxia.

### Measurements

ECG was recorded using a standard electrocardiograph. Systolic (SBP) and diastolic blood pressure (DBP), along with HR, were measured using continuous finger-pulse photoplethysmography (Finometer, Finapres Medical Systems, Arnhem, the Netherlands). Finger blood pressure was calibrated against brachial arterial pressure. Oxyhaemoglobin saturation (SaO_2_) was measured continuously (Massimo Oximeter, Massimo, Milan, Italy) with an ear-clip sensor. Expired air was sampled from the mouthpiece and the end-tidal CO_2_ (EtCO_2_) was measured using a gas analyser (PNT Digital M.E.C. Group, Brussels, Belgium). Doppler ultrasound of the internal carotid artery was performed (*Vivid 7*, GE Healthcare; Little Chalfont, UK) to assess the mean cerebral blood flow velocity (CBFV), pulsatility index (PI) and resistive index (RI). The carotid blood flow was measured in left internal carotid artery 2 cm after bifurcation. All volunteers were free of carotid arteries pathologies (intima-media thickness within the normal range, and no plaques were present) which was documented prior to the experiments.

**Fig 2:**
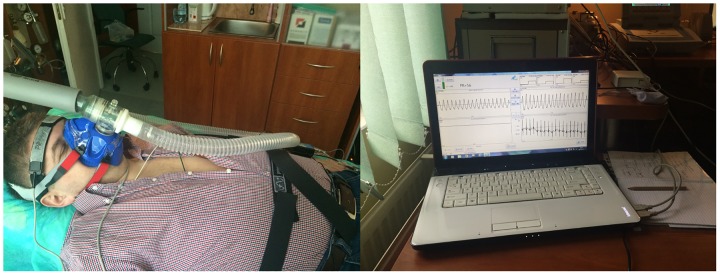
NIR-T/BSS sensors are located in the frontal part of the left and right hemispheres (on the right side). Typical NIR-T/BSS waveforms from both hemispheres are visible on the monitor screen (on the left side). The individual has provided written consent for the use of his image.

The SAS width was recorded with the head-mounted sensors of the NIR-T/BSS device (SAS 100 Monitor, NIRTI SA, Wierzbice, Poland) located in the frontal part of right and left hemispheres (Fig 2). The theoretical and practical foundations of the NIR-T/BSS method were described in earlier studies [15,16]. Briefly, the head-mounted NIR-T/BSS sensor consists of the emitter and two photo-sensors located at various distances from the emitter. A stream of near infrared light generated by the emitter penetrates the highly perfused layer of the skin of the head, the skull bones and the SAS, and the signal is received by the sensors. After the elimination of skin and bone signal absorption, the quotient of the received signals, hereafter called the transillumination quotient (TQ), is sensitive to changes in the width of the SAS. TQ and BP signals are further analysed with wavelet transform method in time-frequency domain. Using empirical mode decomposition, two main components of TQ are identified: the fast (2.0–0.5 Hz) heart-generated component (cc-TQ) and the slow (less than 0.5 Hz) non-cardiac component (sas-TQ). cc-TQ reflects cerebrovascular pulsatility [15,21]. sas-TQ is related to changes in cerebral blood volume [18–20]. cc-TQ and sas-TQ are reported in time domain as arbitrary units.

BP and SAS signals were acquired at 70 Hz via a data acquisition unit (Powerlab/16SP ML795, AD Instruments). All variables were recorded continuously or videotaped, and the signals were routed to a hard drive for further analyses.

### Wavelet transform

Wavelet transform is a method that transforms a time signal from the time domain to the time-frequency domain [29,30]. Wavelet coherence (WCO) was used to determine the amplitude coherence of the wavelet cross-spectrum in the time-frequency domain [31]. A value close to 1 indicates a linear relationship between the two time series as well as a high signal to noise ratio. A value close to zero implies no correlation. Wavelet phase coherence (WPCO) identifies possible relationships by evaluating the match between the instantaneous phases of two signals [29]. The value of the phase coherence *C*_*φ*_(*f*_*k*_) is between 0 and 1. This function quantifies the tendency of the phase difference between the two signals to remain constant at a particular frequency [29]. When two oscillations are unrelated, their phase difference continuously changes with time; thus, their phase coherence approaches zero. As typically some noise (or the effect of exogenous variables) is present in physiological signals, obtaining a value exactly equal to one is rather unlikely. Also nonlinear relations between two signals and/or the effect of additional unobservable variables tend to decrease coherence values.

### Statistical analysis

The Wilcoxon test was used to compare the changes in WCO, WPCO, the cardiac (0.5–5.0 Hz) and slow (<0.5 Hz) components of SAS, SBP, DBP, HR, CBFV, RI, PI, SaO_2_ and EtCO_2_ in response to hypoxia. The sample size was determined on the basis of test power analysis (20 individuals had a power greater than 80% to detect the assumed pial artery pulsation and the width of subarachnoid space changes).

WCO and WPCO assessment was performed with all-time series and generated corresponding surrogate time series. To generated surrogates we used iterative amplitude adjusted Fourier transform—IAAFT [32]. Original WCO and WPCO located above the surrogate WCO and WPCO are considered statistically significant.

## Results

Subjects were effectively down-titrated to reach a blood oxygen saturation of 79.05 ± 3.8% and 79.2 ± 3.7% in the first and fifth minutes of hypoxic challenge, respectively (Table 2).

**Table 2:** Effects of hypoxia on SAS, SBP, DBP, HR, CBFV, PI, RI and SaO_2_. Data are presented as mean values and standard deviations (SD). All % changes are calculated with reference to baseline values.

	Baseline	1 minute	2 minute	3 minute	4 minute	5 minute	1 min recovery	2 min recovery	3 min recovery
cc-TQR (AU)	165.0±77.4	183.1±108.4^NS^	197.6±109.4^NS^	199.4±166.6^NS^	192.7±165.5^NS^	184.7±120.1^NS^	191.7±96.9^NS^	190.5±134.5^NS^	149.1±103.3^NS^
cc-TQL (AU)	95.2±61.0	124.7±85.9^NS^	117.0±84.9^NS^	127.1±90.0^NS^	121.1±106.8^NS^	120.5±86.5^NS^	126.6±79.2^NS^	127.0±85.4^NS^	116.0±95.0^NS^
sas-TQR (AU)	880.4±252.2	818.3±271.4^NS^	819.3±293.5^NS^	824.0±281.8^NS^	823.1±274.4^NS^	777.8±289.7^NS^	820.2±310.6^NS^	822.6±296.7^NS^	855.2±253.4^NS^
sas-TQL (AU)	612.4±161.0	573.2±156.6^NS^	602.2±157.8^NS^	609.9±156.3^NS^	576.8±162.6^NS^	580.6±167.2^NS^	611.1±171.4^NS^	616.6±162.8^NS^	615.0±158.9^NS^
SBP (mmHg)	116.25±7.83	141.95±18.0***	143.85±13.23***	144.1±10.85***	145.2±11.75***	139.6±10.75***	130.8±11.18***	128.7±11.46***	116.9±8.38^NS^
DBP (mmHg)	73.15±8.92	83.85±12.74*	81.6±10.34*	81.2±9.07*	81.65±7.46**	80.6±6.39**	79±7.09*	76.6±6.58^NS^	70.95±7.14^NS^
HR (beats*sec^-1^)	66.85±9.59	84.85±10.76***	85.7±11.76***	79.4±21.74**	80.95±10.85***	77.9±9.9***	65.35±8.42^NS^	63.55±10.32^NS^	64.1±8.96^NS^
CBFV (cm*sec^-1^)	40.96±9.31	41.57±10.2^NS^	42.65±9.26^NS^	43.06±10.04^NS^	44.55±8.57*	43.74±7.64*	40.63±9.54^NS^	37.48±6.34^NS^	37.05±8.52*
RI	0.63±0.12	0.66±0.14^NS^	0.65±0.09^NS^	0.67±0.08^NS^	0.65±0.08^NS^	0.66±0.21^NS^	0.66±0.1^NS^	0.68±0.09^NS^	0.65±0.11^NS^
PI	1.59±0.54	1.64±0.48^NS^	1.53±0.36^NS^	1.54±0.42^NS^	1.37±0.38^NS^	1.32±0.45^NS^	1.46±0.42^NS^	1.48±0.44^NS^	1.42±0.29^NS^
SaO_2_ (%)	97.08±1.68	79.09±3.66***	79.73±2.65***	78.68±3.2***	79.05±3.83***	79.2±3.73***	94.06±2.74***	96.41±1.52*	96.95±1.39^NS^
EtCO_2_ (mmHg)	35±3.53	33.8±3.18*	33.41±3.63*	33.84±2.98^NS^	32.96±3.52**	33.23±3.34*	32.99±3.93^NS^	33.01±3.81*	33.75±3.25^NS^

* P<0.05;

** P<0.01;

*** P<0.001;

cc-TQ—cardiac component of the subarachnoid width (heart-generated pial artery pulsation, from 0.5 Hz to 5.0 Hz); sas-TQ—slow component of the subarachnoid width (<0.05 Hz); SBP—systolic blood pressure; DBP—diastolic blood pressure; EtCO_2_—end-tidal CO_2_; HR—heart rate; MBP—mean blood pressure; CBFV—cerebral blood flow velocity; PI—pulsatility index; RI—resistive index; SaO_2_—oxyhaemoglobin saturation; AU—arbitrary units; mm Hg—millimeters of mercury; s—seconds; L—left hemisphere; R—right hemisphere

Gradual increases in SBP, DBP and HR, the hallmarks of sympathetic activation, were observed immediately after initiating the hypoxic challenge. HR recovered in the first minute and DBP in the second minute after restoration of normal breathing. However, DBP remained elevated even at the third minute after restoration of normal breathing. The decrease in ETCO_2_ suggests hyperventilation, another indicator of sympathetic overactivity (Table 2).

The SAS did not change during five minutes of exposure to hypoxia. CBFV was relatively stable, but a modest increase was observed in the last two minutes of deoxygenation. Internal carotid artery Doppler ultrasound measurements showed no change in either pulsatility or resistive indices throughout the entire experiment (P = NS for all comparisons vs. baseline). However, the CBFV declined at the end of the recovery phase by approximately 4 cm*sec^-1^ (Table 2).

**Fig 3:**
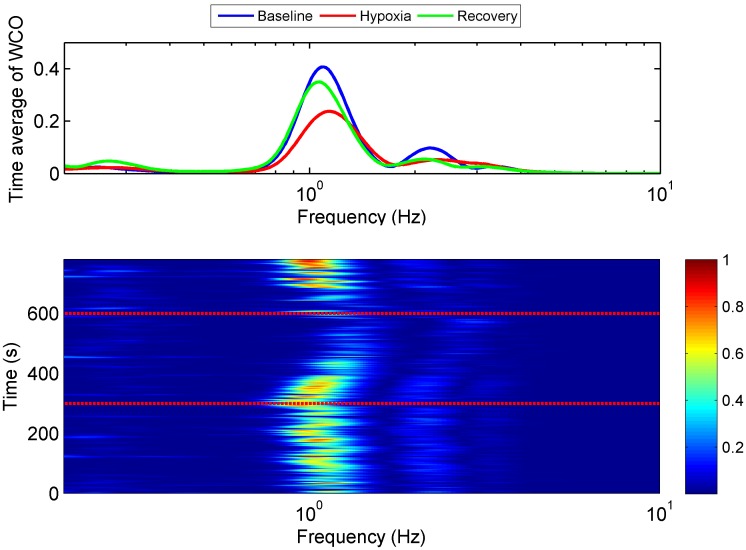
Representative BP SAS wavelet amplitude coherence (WCO) tracings from one subject. Time-averaged WCOs at baseline (blue line), during exposure to hypoxia (red line) and recovery (green line) are shown in the upper panel. Hypoxia results in a decline in WCO. In the lower panel, WCO changes over time are shown. Red horizontal dotted lines indicate the beginning and end of hypoxia exposure (300 s to 600 s). WCO declined after exposure to hypoxia, and later recovered upon restoration of normal breathing.

The interrelationship between cardiac-dependent BP changes and SAS amplitudes oscillations (WCO) diminished immediately after exposure to normobaric hypoxia and recovered within 1–2 minutes after restoration of air breathing (Table 3, Fig 3), whereas wavelet phase coherence did not change throughout the experiment (Table 3). Taken together, the coupling of amplitudes of BP and SAS oscillations became less tight as referred to baseline.

**Table 3:** Effects of a 5 minutes hypoxia and recovery on WCO and WPCO between BP and SAS oscillations at cardiac frequency. Data are presented as mean values and standard deviations (SD).

	Baseline	1 min	2 min	3 min	4 min	5 min	1 Rec	2 Rec	3 Rec
**WCO left**	0.65±0.21	0.49±0.13*	0.49±0.19*	0.41±0.16**	0.46±0.20**	0.47±0.18*	0.51±0.26*	0.61±0.22^NS^	0.61±0.17^NS^
**WCO right**	0.61±0.22	0.46±0.16*	0.47±0.21*	0.46±0.19*	0.42±0.17*	0.45±0.19*	0.56±0.27^NS^	0.57±0.24^NS^	0.59±0.19^NS^
**WPCO left**	0.60±0.50	0.54±0.45^NS^	0.53±0.44^NS^	0.48±0.45^NS^	0.52±0.43^NS^	0.53±0.44^NS^	0.58±0.42^NS^	0.47±0.49^NS^	0.55±0.48^NS^
**WPCO right**	0.63±0.44^NS^	0.62±0.33^NS^	0.60±0.41^NS^	0.67±0.31^NS^	0.49±0.36^NS^	0.53±0.39^NS^	0.64±0.39^NS^	0.51±0.43^NS^	0.57±0.46^NS^

* P<0.05;

** P<0.01;

*** P<0.001;

WCO—wavelet coherence; WPCO—wavelet phase coherence; left—left hemisphere; right—right hemisphere; SD—standard deviation

**Fig 4:**
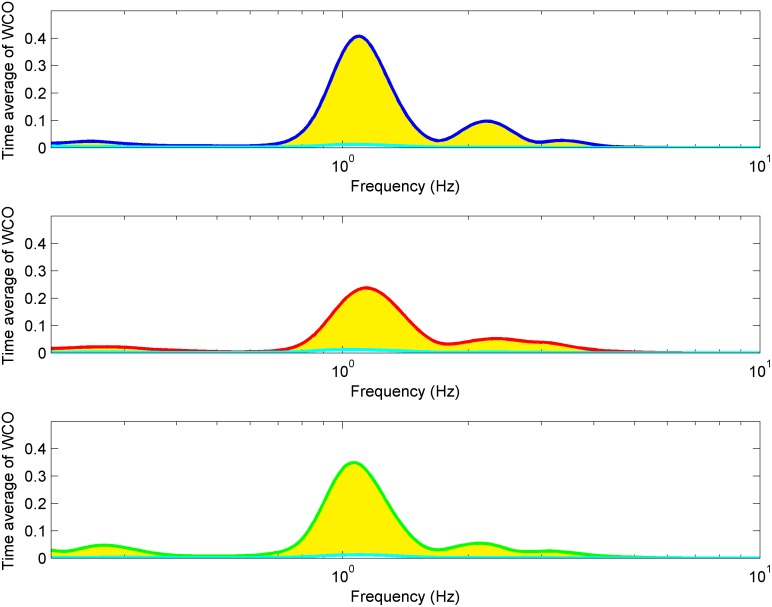
Time-averaged BP-SAS wavelet amplitude coherences (WCOs) from one subject at baseline (top panel), during exposure to hypoxia (middle panel) and recovery (bottom panel). Cyan lines correspond to time average of WCO calculated for surrogate time series estimated from analysed signals. Shaded yellow areas indicate significant WCO.

All-time series are showed on Fig 4. Cyan solid lines illustrate results obtained for IAAFT surrogates [33]. Yellow shaded areas indicate significant coherence [33]. Magenta dashed lines show cardiac frequency. All of them are located in areas of significant amplitude coherence. Similar behaviour we observed for higher harmonics.

## Discussion

Our study shows that: 1) cerebral haemodynamics are relatively stable during very short exposure to hypoxia, 2) changes in cardiac haemodynamics directly affect the coupling between heart-generated blood pressure and subarachnoid space oscillations.

Recent data suggest that an alteration in cardiac output either acutely or chronically changes CBF [34,35]. The mechanisms linking cardiac output fluctuations with brain haemodynamics remain largely unrecognised. The transmission of pulse waves throughout the intracranial environment are related to complex interactions between vascular stiffness and intracranial compliance [36].

In this study, we propose a model where cerebral haemodynamics are relatively stable, as indicated by unchanged SAS, PI and RI indices and only a modest increase (less than 10%) in CBFV by the end of the hypoxic exposure period. Additionally, the observed BP fluctuations were recorded within the “autoregulatory range” at any time-point of the experiment. Surprisingly, immediately after the initiation of hypoxia, we noticed a marked attenuation in amplitude coherence between BP and SAS which indicate reduced coupling between oscillations. Thus, acute hypoxia affects cerebral haemodynamics directly, as no significant changes in other key parameters were evident. Previous experiments have shown that acute hypoxia also does not affect left ventricle stroke volume, so tachycardia appears to be the main driver of cardiac output augmentation [1]. Further studies are warranted to test whether the wavelet coherence between BP and SAS signals at cardiac frequency are sufficiently sensitive to predict any clinical alterations. Commonly encountered diseases such as heart failure or obstructive sleep apnoea are naturally associated with either chronic or intermittent hypoxaemia and hypoxia, the condition which we reproduced in our experiment. Potentially, any novel, especially non-invasive method which may be useful in assessing disease prognosis would be of clinical value.

We used wavelet transform analysis as it ensures windows of adjustable lengths, thereby providing the benefit of showing high resolution at cardiac frequency. Compared with autoregressive estimation, wavelet transform is calculated directly from the data, and the limitations of linear modelling and the choice of model order are thus avoided [37]. The Fourier transform assumes a stationary signal, i.e. that the frequency content does not change over time. Naturally, in biomedical signals, this is never the case, due to the openness of the system, which results in time-varying frequencies of oscillation. One might have divided these frequency intervals into a set of intervals and used short-term Fourier transform with a different window length for each interval. This is actually the same idea as the one that initiated the wavelet transform. But, in the case of the continuous wavelet transform, the number of intervals is no longer finite and the frequency resolution correspondingly changes continuously. The method has already been used by us and others [21–23,29,30,36–38].

As expected, five minutes of exposure to hypoxia was too short to evoke significant changes in SAS in the time domain. This suggests that ICP [39] and SAS [20] swings observed during apnoeic episodes depend rather on CO_2_ accumulation and thoracic pressure swings than decreased brain oxygenation. In this study, we investigated the effect of a poikilocapnic hypoxic challenge where the EtCO_2_ content fluctuated freely and decreased during hypoxia due to reflex hyperventilation, contrary to isocapnic hypoxia where CO_2_ is titrated to account for wash out of CO_2_ resulting from hyperventilation. The EtCO_2_ decline most likely resulted in stabilisation of the CBFV and SAS. However, moderate hyperventilation due to enhanced sympathetic drive does not seem to affect the relationship between BP and SAS oscillations at cardiac frequency [23]. Our results are in line with earlier studies which showed that only prolonged hypoxia increases CBFV [2,40,41]. This phenomenon was evident starting from the fourth minute of our procedure. In a different protocol that mimicked obstructive sleep apnoea episodes, we have shown that CBFV elevation precedes SAS decline [20]. Moreover, the observed trend toward SAS reduction in the present study suggests a further decline in SAS over time.

The results of our study should be put in a wider context. Until now, in our previous research we separately tested the effects of various stimuli typically seen in OSA such as apnoea, negative thoracic pressure and increased sympathetic drive on CSF dynamics ([21–23], PONE-D-16-33950). We have shown that apnoea lasting 90 seconds affects heart-driven BP and SAS amplitude coupling which itself may result in alteration of CSF circulation [21]. Since, apnoea itself is naturally associated with several stimuli at one time such as hypercapnia, hypoxia and autonomic nervous system excitation [42,43] it is difficult to separately estimate net impact of these components on CSF dynamics. There is a suggestion that increases in sympathetic drive alone paradoxically stabilize BP changes—SAS amplitudes coupling [22,23]. Thus the observed uncoupling is mainly driven by hypoxia and negative thoracic pressure [PONE-D-16-33950]. As the experiments were performed in healthy volunteers, our results require further verification in subjects with long-standing OSA as adaptive mechanisms might be implicated.

The method we used to induce hypoxia was influenced by the ventilatory drive which was individual for studied subjects. However, as the participants were clearly instructed not to rapidly change their respiration pattern, the steady-state hypoxia was not difficult to attain and maintain via manual titration of the gas mixtures (increasing amount of atmospheric nitrogen added to the room-air). The latency was no longer than one minute, and the standard deviation of the highest mean value of target blood oxygen saturation was as low as 3.83.

Owing to fact that the NIR-T/BSS methodology has been introduced to the clinical setting only recently, there are several issues which should be underlined. It has been demonstrated that NIR-T/BSS and magnetic resonance imaging are comparable and equivalent modalities for the measurement of SAS [17]. NIRT/BSS direct within-individual comparisons yield excellent reproducibility and repeatability and are therefore reliable [44]. On the contrary, measurements with the use of infrared light do not allow for direct data comparisons between subjects due to differences in skull bone parameters [15,45]. This methodological limitation, however, was irrelevant to our study design as we compared variables within the same subjects recorded at different time points.

Finally, the limitations of the mathematical model used should be acknowledged. Besides many advantages of wavelet transform compared to other analytical approaches, it has some drawbacks. First, wavelet coefficients oscillate with positive and negative values around the singularities, which may complicate their detection and modelling. Second, if input signal is shifted in time or space then wavelet coefficients of the wavelet transform will be changed. Third, aliasing can appear because wavelet coefficients are calculated using iterative time discrete operations with the non-ideal high and low pass filters [46].

## Conclusions

To conclude, there are two main findings in our study that we would like to highlight. For the first time, we have demonstrated that cerebral haemodynamics remain relatively stable during a very short exposure to poikilocapnic hypoxia. Second, changes in cardiac performance directly affect oscillations in the subarachnoid space width. Abnormal subarachnoid width oscillations suggest a disturbed cerebrospinal fluid pulsatility pattern. Our data justify larger scale clinical studies to establish a potential link between oscillatory markers, therapeutic management and disease prognosis.
